# Age disparity in couples and the sexual and general health of the male partner

**DOI:** 10.1111/andr.13738

**Published:** 2024-08-19

**Authors:** Clotilde Sparano, Giulia Rastrelli, Giovanni Corona, Linda Vignozzi, Daniele Vignoli, Mario Maggi

**Affiliations:** ^1^ Endocrinology Unit “Mario Serio” Department of Experimental and Clinical Biomedical Sciences University of Florence Florence Italy; ^2^ Andrology Women's Endocrinology and Gender Incongruence Unit "Mario Serio" Department of Experimental and Clinical Biomedical Sciences University of Florence Florence Italy; ^3^ Endocrinology Unit Azienda AUSL Maggiore Hospital Bologna Italy; ^4^ Department of Statistics Computer Science Applications University of Florence Florence Italy

**Keywords:** ageing, cardiovascular disease, erectile dysfunction, life style, testosterone

## Abstract

**Introduction:**

Several robust epidemiological studies suggest that men are often engaged in sexual relationships with younger women with a variable, age‐dependent age difference. However, the ageing process determines a significant worsening of the andrological status, which favors the onset of erectile dysfunction and hypogonadism.

**Objectives:**

To analyze the effects of differences in age between the partners [delta (Δ) age (*M* − *F*)] on patients referring to the Andrology Unit of Careggi University Hospital for male sexual dysfunction.

**Materials and methods:**

A monocentre cohort of 4055 male subjects was evaluated by SIEDY structured interview. The cross‐sectional analysis assessed the psychobiological and relational correlates. The rate of forthcoming major cardiovascular events (MACE) was investigated in the longitudinal analysis. All the models have been adjusted for age, education, lifestyle, and chronic disease score.

**Results:**

∆ age (M–F) shows a stepwise increase, according to the increasing age bands of the male partner. ∆ age (M–F) was associated with a greater number of children, at the cost of more conflictual relationships within the family. The phenotype of these relationships is characterized by the report of a partner with a higher sexual desire and a higher ability to reach climax. Men seeking a younger partner show more often a histrionic personality (*p* = 0.023) and higher testosterone levels (*p* = 0.032). However, having a younger partner doesn't improve the ability to obtain a full erection. Kaplan–Maier analysis of a longitudinal subgroup of patients followed longitudinally (*N* = 1402) for 4.3 ± 2,59 years, showed that patients in the fourth quartile had a higher rate of forthcoming MACE versus those in the first quartile (*p* = 0.005).

**Discussion and conclusion:**

In subjects with sexual dysfunctions (as in the general population) age‐different relationships increase as a function of male ageing. A greater Δ age (M–F) is associated with specific men and relationship features and a higher risk of MACE.

## 1 INTRODUCTION

Relationships between men and women with age disparities are well‐known couple stereotypes, especially in the context of older men and younger women.^1^ Nonetheless, there is growing evidence of biological downsides from unions with older males.^2^ Historically, marriages between age‐asymmetrical partners were the preferred model due to the reciprocal advantages in terms of economic support (by older men, who were socially accepted as working partners) and fertility (by younger and healthy women, who were considered caretakers of “the hearth”).^3^ Females who marry higher‐status mates and males who choose younger mates have more often surviving children than those following alternative mating strategies.^3^ While the modern era has somewhat mitigated various social predispositions, mutual interests—such as sexual attraction, fertility, and economic well‐being—continue to act as primary motivating factors for many couples with age disparities, often transcending individual predispositions. From a gender perspective, men are still inclined to weave relationships mostly based on physical attraction, at odds with women.^4^ This behavior explains the failure of marriages where husbands progressively lose their partners’ sexual allurement^3^, ^5^ and how ageing adversely affects couples’ stability—fostering contemporary research on the so‐called “grey divorces” or “silver splits.”^6^, ^7^, ^8^ Although the emotional and physical gaps between genders widen over time, currently this trend has resulted in an increase in relationships between individuals from different generations, frequently observed in the context of remarriages or extramarital relationships.^5^, ^9^, ^10^, ^11^, ^12^ Research conducted by Schwarz and Hassebrauck^1^ has demonstrated that as men age, they increasingly gravitate toward younger female partners, often with age disparities of 10 years or more. In the USA, the age gap is 3, 5, and 8 years in the first, second, and third marriages, respectively.^4^

The examination of couples with age disparities has primarily focused on social and psychological dimensions,^13^, ^14^, ^15^ while research into the physio‐pathological impacts on male partners has received limited attention. The ageing process determines a significant worsening of men's overall health.^16^ Accordingly, data from the European Male Aging Study show that men suffer from an age‐dependent progressive decline in both general and sexual health.^17^ Erectile dysfunction (ED) is a common disease that affects about 30% of males but increases to 64% around 80 years old.^18^ In older men, this condition represents not only a sexual symptom but also a cardiovascular (CV) warning, which increases by a factor of 2 the mortality risk, regardless of the hormonal status.^19^, ^20^ On the other hand, testosterone levels are pivotal for sexual and general health. Testosterone exerts its action on the whole body, including CV and musculoskeletal systems, bones, metabolic profile, mental health, and cognitive abilities.^20^, ^21^, ^22^, ^23^

As men age, testosterone levels typically experience a gradual decline, often attributed to age‐related morbidities that predispose individuals to primary and secondary hypogonadism.^24^, ^25^ Given the projected significant increase in the older adult population, it is crucial to thoroughly examine the potential relationship between andrological status and partnerships with younger individuals, especially regarding their effects on sexual and general health. This study aims to examine the clinical, hormonal, and pathological variances among men seeking andrological care, with a focus on the age of their partners and the age disparity within the couple.

## 2 METHODS

### 2.1 Cross‐sectional analysis

A consecutive series of 4055 men referred to the Andrology Unit of Careggi University Hospital of Florence from 2002 to 2015, has been considered for this study. Educational level, lifestyle (alcohol and smoking) habits, and number of children have been collected through specific questions as previously reported.^26^, ^27^, ^28^ The psychobiological and relational correlates were assessed by the structured interview on erectile dysfunction (SIEDY),^29^ a validated 13‐item questionnaire. Briefly, SIEDY is composed of three different scales, estimating different facets of male sexual dysfunction: Scale 1 deals with the organic domain (questions #4, #13, and #15), while Scales 2 (questions #7, #8, #9, and #10) and 3 (questions #2, #3, #6, #11, #12, and #14) concern the relational^9^ and intrapsychic^30^ domains, respectively. Regarding couple relationships, each patient was interviewed about the partner's age, relationship stability (#5), conflict with the partner (#6) or within the family (#11), partner's desire (#8) and the ability to reach climax (#9), partner's health status (#7), menopause (#10) and eventual other sexual relationships (#15).

The characteristics of ED were assessed using SIEDY Appendix A, as previously described.^29^

To better explore the psychological symptomatology of each patient, the Middlesex Hospital Questionnaire (MHQ), which screens potential mental disorders in a non‐psychiatric setting^31^ was also utilized. This test explores different psychiatric domains including free‐floating (MHQ‐A) or phobic anxiety (MHQ‐P), obsessive behaviors (MHQ‐O), somatization (MHQ‐S) or depressive (MHQ‐D) symptoms, and histrionic personality (MHQ‐H). Of note, the total score of MHQ(∑MHQ) identified the psychological attitude and the personality of each investigated subject.^9^

Each patient underwent a complete clinical and blood test examination. The main clinical data were: weight (kg) and height (cm) to calculate the body‐mass index (BMI) (kg /m^2^); waist (cm); testis volume (mL); and arterial blood pressure (mmHg). The impact of concurrent comorbidities was calculated from the patient's medical history and drugs and its value was estimated by the chronic disease score (CDS).^32^ Each patient was also scored with different CV risk engines, including Framingham,^33^ PROCAM,^34^ and Rischio Cuore, specifically designed for the Italian population.^35^

Each patient underwent a full biochemical and hormonal screening. Blood samples were drawn in the morning, after an overnight fast, for determination of glucose (esokinase method; Dimension Vista 1500 Medical Solutions by Siemens Healthcare, Newark, USA) and glycated hemoglobin (HbA1c) (high prestation liquid chromatography, HPLC, Variant II method, Biorad Laboratories, Hercules, CA, USA); total cholesterol, high‐density lipoprotein (HDL) and triglycerides (automatic enzymatic colorimetric method; Dimension Vista 1500 Medical Solutions by Siemens Healthcare, Newark, USA); total testosterone (TT), and prolactin (PRL) levels (by electrochemiluminescent method; Roche, Milan, Italy); sex hormone binding globulin (SHBG) (using the electrochemiluminescence immunoassay; COBAS, ROCHE, Germany); thyroid‐stimulating hormone (TSH), follicle‐stimulating hormone (FSH), luteinizing hormone (LH). Low‐density lipoprotein cholesterol was calculated (LDLc) with the Friedewald equation [LDL cholesterol  =  total cholesterol – (HDL cholesterol + triglycerides/5)].

Penile color‐Doppler ultrasound (PDCU) was performed at the Andrology Unit of Careggi Hospital in 1747 cases, and the results of the peak of systolic velocity in flaccid conditions (basal; bPSV) were collected. Of them, 1676 also underwent a dynamic assessment after an intracavernous injection of 10 µg of prostaglandin E_1_ to determine the dynamic PSV (dPSV) as previously reported.^36^, ^37^ The characteristics of these subsets did not differ from those observed in the whole sample (not shown). All the data provided were collected as part of the routine clinical procedure, according to our hospital's approval protocol (L99‐A08 292/2014) for the diagnostic workup for each patient referred to our unit for sexual dysfunction.

### 2.2 Longitudinal analysis

A subgroup of 1402 patients, followed longitudinally for 4.3 ± 2.59 years was evaluated to assess the occurrence of major adverse cardiovascular events (MACE) as previously reported.^38^, ^39^ The characteristics of this sample did not differ from those derived from the whole population. To verify the relationship between couples with age differences and CV disease, we also applied two other non‐conventional CV risk factors, such as the patient's reported female sexual desire^37^ and PCDU‐assessed penile blood flow.^36^

### 2.3 Statistical analysis

Continuous variables were expressed as mean ± standard deviation (SD) or median [interquartile range (IQR)] when normally or non‐normally distributed, respectively. Categorical variables were expressed as percentages. The difference in age between male and female partners [delta (Δ) age (M–F)] was calculated as a positive value, meaning the male partner is older than the female one; conversely, a negative value means the female partner is older than the male one. Greater values, which denote a wider age difference between the partners, have been used both as a continuous variable and as quartiles. Kruskal–Wallis, or ANOVA, was performed to compare groups according to sample size and distribution.

In the longitudinal analysis, Kaplan–Meier curves were used to calculate survival‐free from MACE in the whole population, and after splitting the sample according to partner desire (present/absent), assessed at the patient's first evaluation. Quartiles of Δ age (M–F) were compared by log‐rank tests.

Regarding the cross‐sectional analysis, fully adjusted (age, education, lifestyle, and CDS) multiple linear regressions were estimated to verify the effect of Δ age (M–F) and other parameters in predicting the following outcomes: number of children, psychobiological symptoms (according to MHQ), testosterone levels, and the basal and maximal peak systolic velocity. Similarly, fully adjusted (age, education, lifestyle, and CDS) multiple binary regressions were performed to verify the effect of Δ age (M–F) on the relationships features, that is: the ability of the partner to reach climax (no/yes), absence of partner hypoactive sexual desire (no/yes), conflict with the family (no/yes), number of intercourses > 8/month (no/yes), relationships lasting > 5years (no/yes) or unstable relationships (no/yes).

Regarding the longitudinal analysis, fully adjusted (age, education, lifestyle, and CDS) Cox‐regression analyses have been performed to assess the risk of forthcoming MACE in different models, including unconventional CV risks, such as maximal dynamic peak systolic velocity and partner libido as further covariates.

All the analyses and figures were performed with IBM SPSS version 26 and GraphPad Prism version 9.

## 3 RESULTS

### 3.1 Cross‐sectional analysis

**FIGURE 1:**
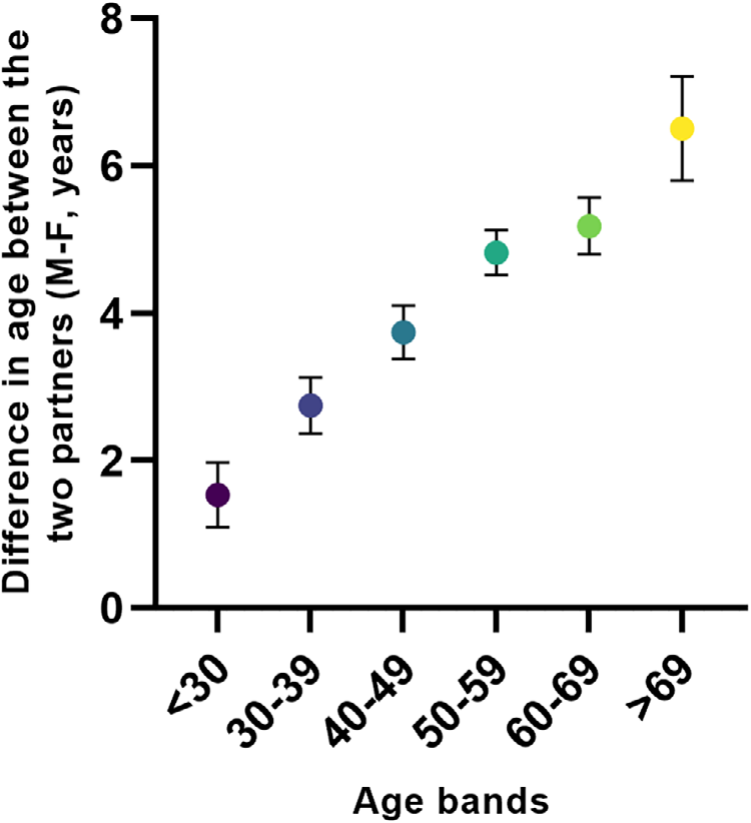
Relationship between male age bands and ∆ age (M–F) in a consecutive series of subjects with sexual dysfunction.

Overall, the patients’ and partners’ mean age was 51.3 ± 13.3 and 48.2 ± 12.7 years old, respectively, with a median Δ age (M–F) of 4 years [1–7]. Δ age (M–F) increases as a function of male age. Figure 1 shows the stepwise increase in Δ age (M–F) according to the increasing age bands of the male partner. In particular, males aged 69 or older showed an average 6‐year difference with the female partner age, while in males aged 30 or younger, the difference was less than 2 years.

**FIGURE 2:**
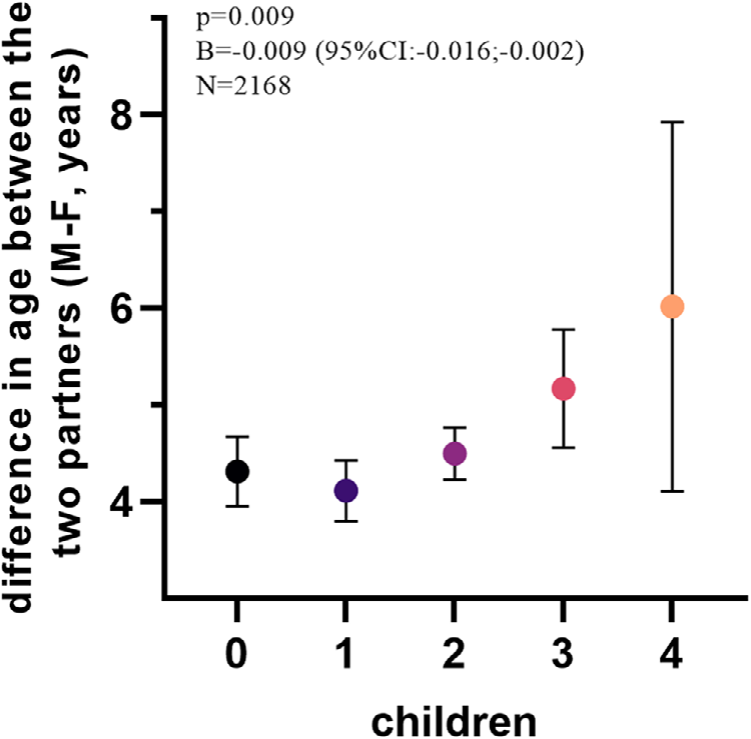
Age‐, CDS‐, education‐ and the lifestyle‐adjusted relationship between the number of children and ∆ age (M–F) in a consecutive series of 2168 subjects with sexual dysfunction. CDS, Chronic disease score.

Table 1 shows descriptive statistics according to Δ age (M–F) quartiles and reports relative differences among quartiles. According to Δ age (M–F) ranges for quartiles, no significant differences were observed among the majority of parameters examined. In linear regression analyses, after adjusting for possible confounders, including male age, education, and lifestyle (drinking and smoking behavior), several of the observed differences in Table 1 did not retain significance. In particular, in the adjusted model, the positive relationship between Δ age (M–F) and CDS (a broader index of associated morbidities) was not confirmed (*p* = 0.436, adjusted *R* = 0.476). After introducing CDS in the aforementioned adjusted model (male age, lifestyle, education), also differences in fasting glycemia, total cholesterol, SHBG, and PSA were not confirmed (not shown). However, Δ age (M–F) was associated with a stepwise increase in the number of children (Figure 2), even in the fully adjusted model (male age, lifestyle, education, and CDS; *N* = 2168). The non‐significant difference in scoring in the major CV risk engines including Framingham, PROCAM, and Rischio Cuore, and Δ age (M–F) was observed (not shown).

**TABLE 1:** Demographic data of Δ age (male–female age) quartiles on 4055 subjects consulting for sexual dysfunction.

			Quartiles of Δ age (M–F)				
			1st	2^nd^	3rd	4th	
Factor			**‐27;1**	**2;3**	**4;6**	**7;43**	
Δ age (M–F) (min; max)	Units		***N* = 1101**	***N* = 842**	***N* = 1097**	***N* = 1015**	*p* ^a^
Graduated	Yes	*n* (%)	337 (42.5)	266 (47.2)	310 (49.2)	296 (45.6)	0.077
Alcohol	<4 units	*n* (%)	1058 (96.6)	813 (96.8)	1050 (96.2)	968 (96.4)	0.885
	>4 units	*n* (%)	37 (3.4)	27 (3.2)	42 (3.8)	36 (3.6)	
Smoke	Yes	*n* (%)	312 (28.4)	226 (27.1)	320 (29.9)	307 (30.9)	0.278
Children	0	*n* (%)	352 (32.7)	187 (22.4)	261 (24.1)	316 (31.8)	**<0.001**
	1	*n* (%)	324 (30.1)	251 (30.1)	296 (27.3)	256 (25.7)	
	2	*n* (%)	328 (30.4)	326 (39.1)	427 (39.4)	316 (31.8)	
	3	*n* (%)	56 (5.2)	55 (6.6)	83 (7.7)	84 (8.4)	
	≥4	*n* (%)	18 (1.7)	15 (1.8)	17 (1.6)	23 (2.3)	
BMI	kg/m^2^	Mean (SD)	26.68 (4.18)	26.68 (4.12)	26.46 (3.99)	26.99 (4.30)	0.053
Waist	cm	Mean (SD)	98.01 (10.86)	97.95 (10.63)	97.44 (10.31)	98.80 (11.03)	0.053
Right testis volume	mL	Mean (SD)	19.30 (4.80)	19.07 (5.02)	19.26 (4.59)	19.04 (4.80)	0.547
Left testis volume		Mean (SD)	18.90 (4.68)	18.68 (4.81)	18.91 (4.41)	18.78 (4.70)	0.678
Systolic blood pressure	mmHg	Median [IQR]	130.00 [120.00, 145.00]	130.00 [120.00, 145.00]	135.00 [125.00, 140.00]	135.00 [125.00, 145.00]	0.158
Diastolic blood pressure		Median [IQR]	80.00 [80.00, 90.00]	80.00 [80.00, 90.00]	80.00 [80.00, 90.00]	80.00 [80.00, 90.00]	0.073
Mean blood pressure		Median [IQR]	100.00 [93.33, 106.67]	100.00 [93.33, 106.67]	100.00 [93.33, 106.67]	100.00 [93.33, 108.33]	0.067
CDS		Median [IQR]	0.00 [0.00,3.00]	0.00 [0.00,4.00]	0.50 [0.00,4.00]	2.00 [0.00,4.00]	**0.001**
Hb	g/dL	Median [IQR]	14.90 [14.10, 15.80]	14.60 [13.60, 15.50]	15.00 [14.30, 15.53]	14.90 [14.00, 15.40]	0.084
Fasting glycemia	g/L	Median [IQR]	100.00 [93.33, 106.67]	0.96 [0.88, 1.08]	0.95 [0.86, 1.10]	0.97 [0.87, 1.12]	**0.010**
Insulin	µUI/mL	Median [IQR]	7.90 [5.52, 10.90]	7.80 [5.59, 13.85]	8.40 [5.50, 13.80]	9.30 [6.30, 14.80]	0.193
HbA1c	%	Mean (SD)	6.90 (1.68)	6.73 (1.53)	6.85 (1.58)	6.80 (1.59)	0.729
Total cholesterol	mg/dL	Mean (SD)	200.78 (41.64)	200.33 (39.83)	206.11 (41.19)	203.61 (39.84)	**0.013**
HDL cholesterol		Median [IQR]	46.00 [40.00, 54.00]	48.00 [41.00, 57.00]	47.00 [41.00, 54.00]	46.00 [40.00, 54.00]	0.082
Triglycerides		Median [IQR]	117.00 [83.00, 169.00]	111.00 [81.00, 155.00]	119.00 [85.00, 166.50]	118.00 [85.00, 168.00]	0.131
Total T	nmol/L	Mean (SD)	15.32 (6.14)	15.30 (6.13)	15.44 (6.24)	15.55 (6.57)	0.825
SHBG		Median [IQR]	30.50 [24.00, 40.00]	32.10 [23.70, 42.95]	33.00 [24.00, 44.25]	35.05 [26.00, 45.62]	**<0.001**
Calculated free T		Median [IQR]	0.29 [0.22, 0.37]	0.29 [0.21, 0.36]	0.28 [0.21, 0.37]	0.27 [0.21, 0.36]	0.171
LH	UI/L	Median [IQR]	3.70 [2.54, 5.28]	3.70 [2.60, 5.42]	3.96 [2.60, 5.60]	3.90 [2.70, 5.85]	0.089
FSH		Median [IQR]	4.72 [3.05, 8.00]	4.54 [3.00, 7.70]	4.61 [3.03, 7.70]	5.00 [3.30, 8.50]	0.088
PSA	ng/mL	Median [IQR]	0.81 [0.50, 1.36]	0.80 [0.51, 1.37]	0.90 [0.57, 1.50]	0.87 [0.54, 1.51]	**0.028**
PRL	mUI/L	Median [IQR]	154.00 [111.15, 222.50]	154.00 [108.75, 222.00]	151.00 [110.00, 219.00]	152.50 [108.00, 214.25]	0.692
TSH	mUI/L	Median [IQR]	1.48 [1.03, 2.15]	1.41 [0.98, 1.99]	1.41 [1.02, 1.98]	1.43 [1.01, 2.00]	0.160
fT4	pmol/L	Median [IQR]	13.29 [11.90, 16.40]	14.00 [11.96, 15.44]	13.95 [11.83, 16.31]	13.80 [11.71, 15.70]	0.715
fT3		Median [IQR]	5.06 [4.43, 5.48]	4.94 [4.50, 5.44]	4.99 [4.52, 5.59]	5.04 [4.45, 5.52]	0.884
MHQ total score		Median [IQR]	28.00 [16.00, 37.00]	28.00 [19.00, 38.00]	28.00 [19.00, 37.00]	29.00 [20.00, 39.00]	0.076

*Note*: Bold numbers highlight *significant* comparisons.

Abbreviations: BMI, Body mass index; CDS, chronic disease score; FSH, follicle stimulating hormone; fT3, free triiodothyronine; fT4, free thyroxine; HDL, density lipoprotein; IQR, interquartile range; LH, luteinizing hormone; max, maximum; MHQ, mental health questionnaire; min, minimum; *n*, number; PRL, prolactin; PSA, prostatic specific antigen; SD, standard deviation; T, testosterone; TSH, thyroid stimulating hormone.

^a^ *p*‐values refer to comparisons among the four groups.

**FIGURE 3:**
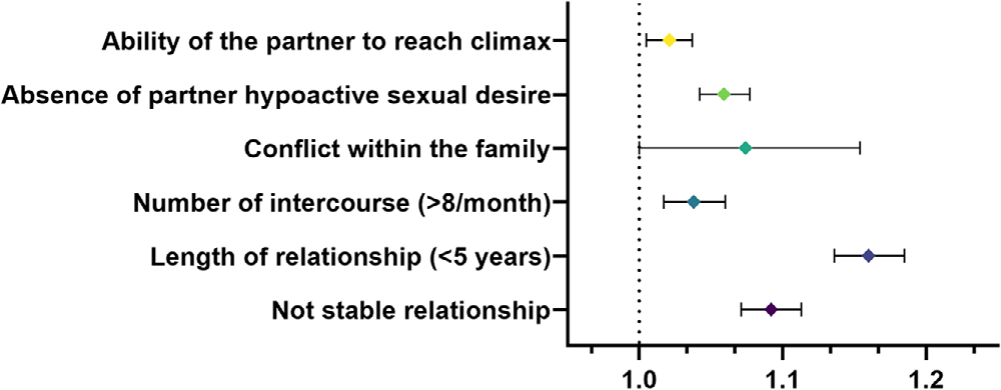
The odds ratio for relationship characteristics, according to increasing ∆ age (M–F) in iterative age‐, CDS‐, education‐ and the lifestyle‐adjusted binary regression models. CDS, Chronic disease score.

In the fully adjusted model (male age, lifestyle, education, CDS), Δ age (M–F) was associated with several factors characterizing the phenotype of the relationship. Having a greater Δ age (M–F) was more often associated with the report of a partner with a higher sexual desire and a higher ability to reach climax (Figure 3). Accordingly, the number of reported intercourses was higher in relationships characterized by a greater Δ age (M–F) (Figure 3). In addition, Δ age (M–F) was also associated with a short‐lasting relationship that was more often unstable and of conflictual dynamics within the family of origin (Figure 3).

**FIGURE 4:**
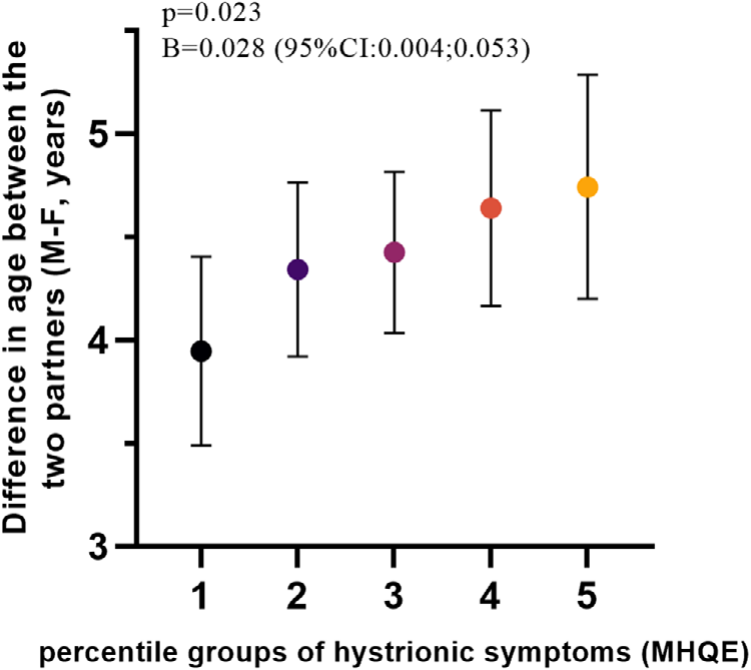
Age‐, CDS‐, education‐ and the lifestyle‐adjusted relationship between the quintiles of histrionic symptoms and ∆ age (M–F), in a consecutive series of subjects with sexual dysfunction. CDS, Chronic disease score.

When psychopathological traits were investigated in the fully adjusted model, we found no differences, according to Δ age (M–F), in free‐floating, phobic, and somatized anxiety, along with depressive and obsessive‐compulsive traits (not shown). In contrast, we found a stepwise positive relationship between increasing Δ age (M–F) and a histrionic personality (*p* = 0.023) (MHQ‐H, Figure 4).

**FIGURE 5:**
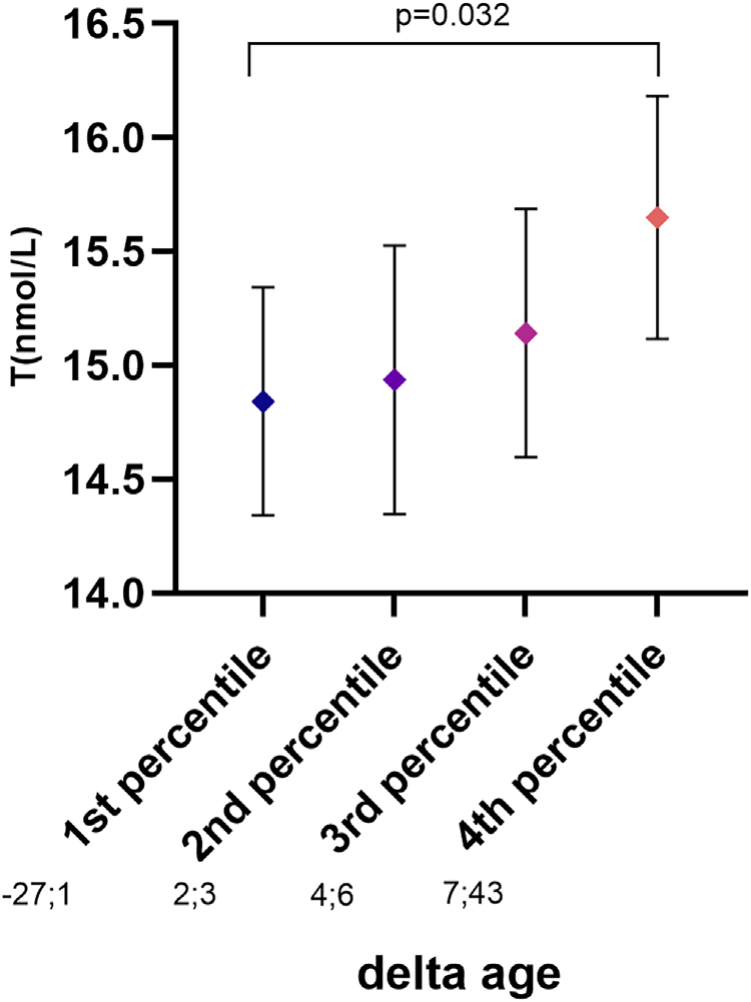
Age‐, CDS‐, education‐ and the lifestyle‐adjusted relationship between quartiles groups of ∆ age (M–F) and total testosterone levels, in a consecutive series of subjects with sexual dysfunction. CDS, Chronic disease score.

In the fully adjusted model, no major difference was found among all investigated hormonal parameters and Δ age (M–F), except for total testosterone, which was higher in the fourth percentile of Δ age (M–F) than in the first one (Figure 5). The association was confirmed even when BMI was introduced in the model as a further covariate (*p* = 0.006) (Table 2).

**TABLE 2:** Age‐, education‐, CDS‐, and lifestyle‐adjusted linear regression analyses of the relationship between Δ age (M–F) and total testosterone levels, without (model 1) and with (model 2) including body‐mass index in the algorithm.

	Model 1 *F *= 14.175, *p* < 0.001			Model 2 *F *= 33.371, *p* < 0.001		
	*B*	95% CI	*p* ^a^	*B*	95% CI	*p* ^a^
Male age	−0.045	−0.07; −0.019	**<0.001**	−0.035	−0.060; −0.011	**0.005**
Education	0.123	−0.178; 0.423	0.423	−0.078	−0.370; 0.214	0.599
Smoking	0.956	0.349; 1.563	**0.002**	0.708	0.117; 1.299	**0.019**
Alcohol	−0.556	−1.050; −0.063	**0.027**	−0.369	−0.851; 0.113	0.134
CDS	−0.253	−0.373; −0.133	**<0.001**	−0.059	−0.179; 0.060	0.329
Δ age (M–F)	0.061	0.015; 0.107	**0.010**	0.062	0.018; 0.107	**0.006**
BMI	–	–	**–**	−0.405	−0.468; −0.341	**<0.001**

*Note*: Bold numbers highlight significant comparisons.

Abbreviations: BMI, Body‐mass index; CDS, chronic disease score; CI, confidence interval; TT, total testosterone.

^a^ *p*‐values refer to comparisons among the four groups.

**FIGURE 6:**
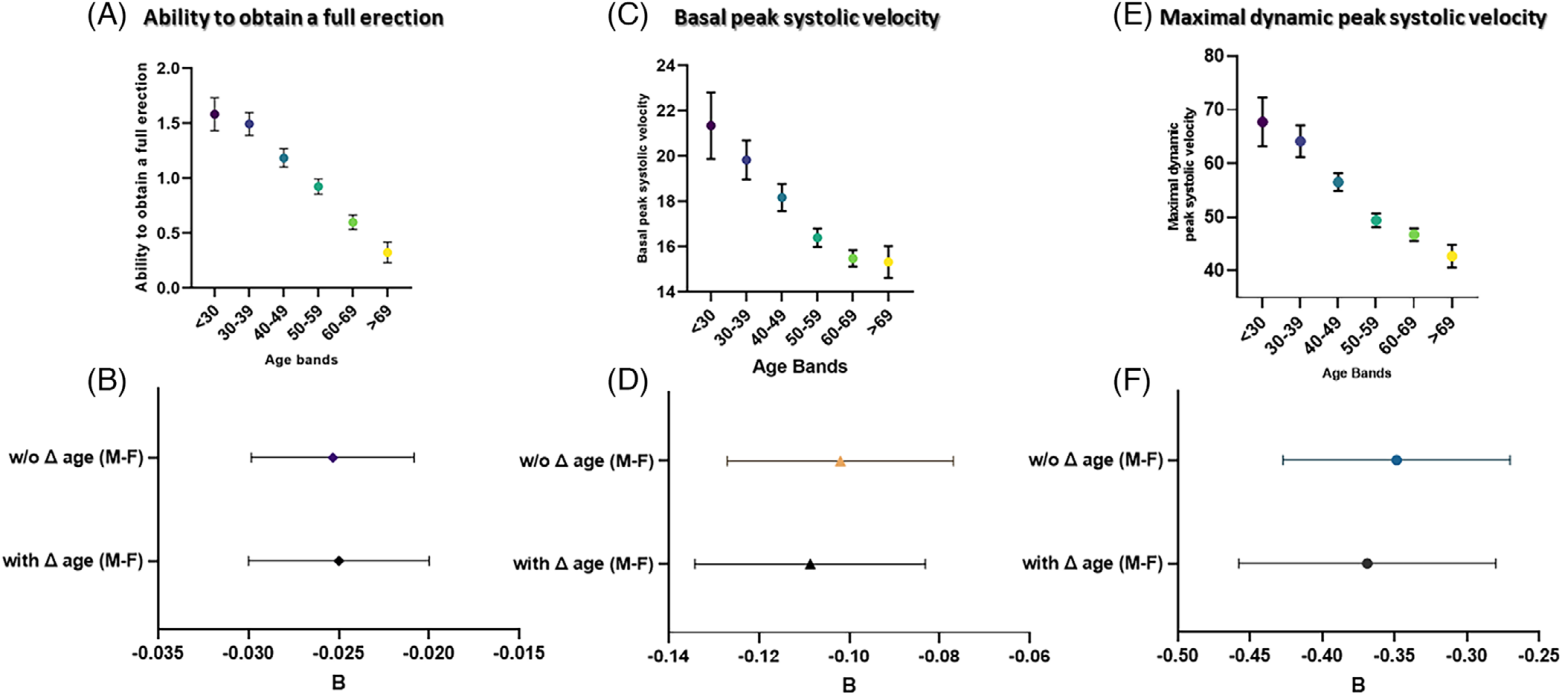
Age‐, CDS‐, education‐ and lifestyle‐adjusted unstandardized B coefficients of erectile function (by SIEDY score) (A), basal (B) and maximal dynamic (C) peak systolic velocity (cm/s^2^) without and with ∆ age (M–F) into the regressions in a consecutive series of the indicated subjects with sexual dysfunction. CDS, Chronic disease score.

The ability to obtain a full erection declines as a function of age (Figure 6A) even after the adjustment for the aforementioned confounders (*p* < 0.001, *B* = −0.025 [−0.03;−0.021]). Figure 6B shows the fully adjusted *B* coefficient and its confidence interval. As shown in Figure 6B, the introduction of Δ age (M–F) in the model did not affect the relationship between increasing age and the inability to obtain a full erection (*p* = 0.325, *B* = −0.025 [−0.03;−0.020]). Similar results were observed when the age‐dependent decline of penile blood flow was considered (Figure 6 C,E). Basal PSV (bPSV, panel C) and maximal dynamic PSV (dPSV, panel E) at PCDU significantly decreased as a function of age (*p* < 0.001, *B* = −0.102 [−0.127; −0.077]; *p* < 0.001, *B* = 0.349 [−0.427;−0.270], respectively), but the introduction of Δ age (M–F) in the model (panels D,F) did not significantly affect the relationship (*p* = 0.454). However, males with a greater Δ age (M–F) experienced less often a total absence of erection, which was confirmed in the fully adjusted model (*p* = 0.048, not shown).

Considering that in the first quartile of Δ age are included couples with positive and negative values of Δ age (M–F) (see Table 1), we performed a sub‐analysis to verify whether a negative Δ age value (i.e., female older than the male partner) is associated with different biological and relational characteristics than those with a positive value. Within the first quartile (*n* = 1101), men with an older partner (*n* = 451) were younger than those without (*n* = 650), with a median age of 48 [39–58] versus 52.5 [40–61.24] years (*p* < 0.001), while female age was not statistically different between groups (52 [40–61] vs. 52 [42–62]). After adjusting for male age, having an older female partner is associated with a lower ability to fatherhood (*p* = 0.005). However, the association did not retain significance after further adjusting for other covariates, including education, lifestyle and CDS (not shown). All the other sexual (erection, desire, ejaculation), biochemical and hormonal parameters were not statistically different between groups (not shown). However, within the first quartile of Δ age, having an older female partner is positively associated with a woman's menopausal state and with a more conflictual relationship, even after adjusting for all the aforementioned confounders (*p* = 0.016 and *p* = 0.02, respectively).

### 3.2 Longitudinal analysis

**FIGURE 7:**
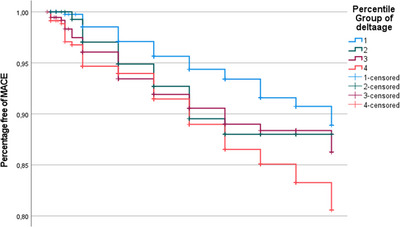
Kaplan–Meier analysis of major cardiovascular events in a subgroup of patients followed longitudinally according to quartiles of Δ age (M–F).

Kaplan–Meier analysis showed a significant difference in forthcoming MACE according to Δ age (M–F) at the study entry (Figure 7). In particular, those in the fourth quartile showed a significantly higher incidence of MACE when compared to those in the first quartile (*p* = 0.005). Cox‐regression analysis confirmed a significantly increased risk of MACE in those with a greater Δ age (M–F), even in the fully adjusted model (OR = 1.245 [CI 95%: 1.005–1.543], *p* = 0.045) (Table 3). The introduction of another risk factor, such as dPSV,^36^ did not attenuate the relationship (OR = 1.435, [CI 95%: 1.087–1.896], *p* = 0.011) (Table 3). Similar results were observed when partner sexual desire was introduced in the previous adjusted model (OR = 1.429, [CI 95%: 1.086–1.880], *p* = 0.011) (Table 3).

**TABLE 3:** Age‐, education‐, CDS‐ and lifestyle‐adjusted Cox regression analyses of the relationship between Δ age (M–F) and forthcoming MACE (Model 1). In Model 2 maximal dynamic peak systolic velocity at PCDU was introduced in Model 1 as a further covariate. In Model 3 partner libido was introduced in Model 2 as a further covariate.

	Model 1			Model 2			Model 3		
	HR	95% CI	*p* ^a^	HR	95% CI	*p* ^a^	HR	95% CI	*p* ^a^
Male age	1.066	1.037–1.096	**<0.001**	1.040	1.003–1.078	**0.035**	1.029	0.991–1.067	0.132
Education	0.797	0.624–1.018	0.069	0.768	0.559–1.054	0.102	0.757	0.544–1.053	0.098
Smoke	1.720	1.020–2.902	**0.042**	1.480	0.765–2.866	0.245	1.453	0.741–2.847	0.276
Alcohol	1.217	0.851–1.740	0.283	1.291	0.872–1.912	0.202	1.219	0.819–1.812	0.329
CDS	1.150	1.046–1.266	**0.004**	1.087	0.956–1.235	0.205	1.089	0.953–1.243	0.210
Δ age (M–F)	1.245	1.005–1.543	**0.045**	1.435	1.087–1.896	**0.011**	1.429	1.086–1.880	**0.011**
Dynamic peak systolic velocity	–	–	**–**	0.981	0.963–0.999	**0.042**	0.980	0.962–0.999	**0.041**
Partner libido	–	–	**–**	–	–	**–**	2.267	1.189–4.324	**0.013**

Abbreviations: CDS, Chronic disease score; CI, confidence interval; HR, hazard ratio; TT, total testosterone.

^a^ *p*‐values refer to comparisons among the four groups.

**FIGURE 8:**
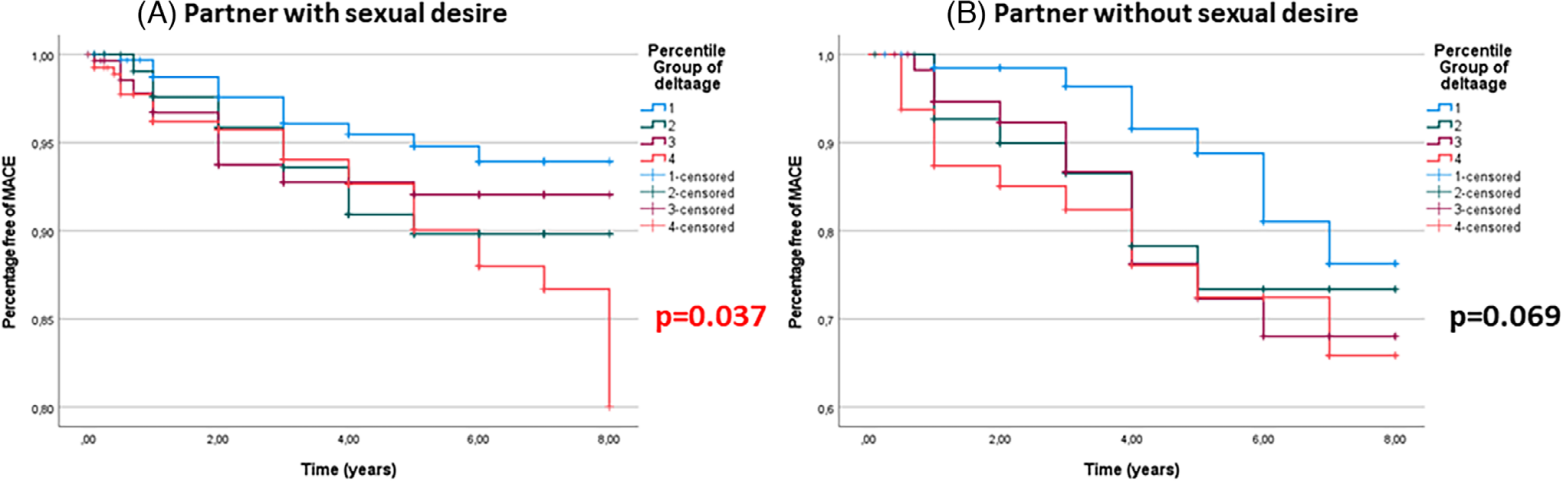
Kaplan–Meier analysis of major cardiovascular events (MACE) in a subgroup of patients according to percentile groups of Δ age (M–F) categorized for partner sexual desire.

In addition, when the entire cohort was categorized according to perceived female sexual desire as a dummy variable (yes/no), the Δ age (M–F)‐associated increased risk was confirmed only in those having a partner with a reported sexual desire (*p* = 0.037), but not in those without (*p* = 0.069) (Figure 8). Fully‐adjusted Cox regression analyses, including dPSV as a further covariate, confirmed the results (Table 4).

**TABLE 4:** Age‐, education‐, CDS‐, and lifestyle adjusted Cox regression model according to the partner's sexual desire.

	HR	95% CI	*p* ^a^
With sexual desire			
Male age	1.030	0.986–1.076	0.182
Education	0.890	0.599–1.323	0.565
Smoke	1.714	0.771–3.812	0.186
Alcohol	1.031	0.557–1910	0.923
CDS	1.166	1.000–1.360	**0.050**
Δ age (M–F)	1.514	1.051–2180	**0.026**
Dynamic peak systolic velocity	0.987	0.966–1.008	0.213
W/O sexual desire			
Male age	1.066	0.994–1.144	0.075
Education	0.441	0.209–0.928	0.031
Smoke	1.797	0.473–6.829	0.389
Alcohol	1.951	1.031–3.691	**0.040**
CDS	0.786	0.570–1.084	0.142
Δ age (M–F)	1.483	0.888–2.478	0.132
Dynamic peak systolic velocity	0.931	0.883–0.983	**0.010**

*Note*: Bold numbers highlight significant comparisons.

Abbreviations: CDS, Chronic disease score; CI, confidence interval; HR, hazard ratio; TT, total testosterone; w/o, without.

^a^ *p*‐values refer to comparisons among the four groups.

## 4 DISCUSSION

Age disparities in couples, with older men and younger women, are a well‐established archetype, involving humans as well as numerous other mammalian species. This study confirms, in a large population of subjects consulting for sexual dysfunction, that older men more often seek a younger partner in an age‐dependent manner.^1^ We here report that a greater age difference [Δ age (M–F)] is associated with a greater chance of having a(nother) child. This finding is in line with similar observations in other cohorts^3^, ^4^ and can be considered as a positive readout for the consistency of the present results, although obtained in a particular population, such as subjects consulting for sexual dysfunctions.

We originally reported that having a younger partner is associated with a more favorable relationship, characterized by a greater number of intercourses with a sexually desiring partner that is more often able to reach climax. However, this relationship is often unstable, of short duration, and not well accepted by the family of origin, making it a source of conflict.^5^, ^9^, ^10^, ^11^, ^12^

Family hassles represent a well‐known stressor for men's sexual health and usually affect relationships’ global functioning by causing mental distress and worsening of ED.^40^ Nonetheless, in couples with increased Δ age (M‐F), these conflicts are not a source of anxiety or depressive symptomatology. The intrapsychic features of male partners can explain this lack of negative psychological repercussions. Men seeking a younger partner often have a histrionic personality and higher testosterone levels. These psychobiological correlates are not surprising, since Bandini et al.^41^ already observed a favorable association between hysterical/histrionic personality and testosterone levels, which underpins better sexual functioning and androgenization. Of note, the histrionic trait belongs to men with seductive ability but also extreme emotional behaviors, and a desire to be in the spotlight, at the cost of being overkill.^41^, ^42^ These psychological features are suited to conquer younger partners and explain the sexual success of their relationship. Of note, higher testosterone levels play an ultimate role in promoting successful mating strategies, favoring a higher rate of intercourse and a low tendency to blame themselves or to have a lack of self‐confidence.^41^

Notwithstanding the above, the age‐dependent decline in the ability to obtain a full erection is not attenuated by having a younger partner, as reported by the patient or objectively measured at PCDU. The erectile function suffers from ageing, and, even with different degrees, ED problems can be found in more than half of the male population after 40 years old, with a growing prevalence.^19^ Furthermore, it has been observed in large male cohorts that the negative effect of ED is independent of the hormonal levels (total of free T levels) and predicts higher mortality rates than in men without ED.^19^ Besides ageing, ED and CV diseases share other common detrimental stressors such as negative recreational habits (i.e., smoking and alcohol), hypertension, and dysmetabolic status.^18^ The vascular health status, measured by penile arterial impairment, serves as an important alert for male CV risk. While it can benefit from improving modifiable risk factors, such as negative habits and metabolic status, the ageing effect cannot be mitigated. In the present cohort, even after correcting for all the main negative risk factors, increasing couples’ Δ age (M–F) does not improve ED. This is tantamount to saying that, despite the better sexual functioning of women with older mates, male partners do not benefit, in terms of ED and penile blood flow, from them. Surprisingly, the CV risk is even higher in men in relationships with younger partners, as derived from the longitudinal analysis. The Kaplan–Meier survival curve indeed shows a significantly different rate of MACE between the first and fourth Δ age (M–F) quartiles, where wider age differences with female partners increase the risk of forthcoming CV events. Even if Δ age (M–F) is not associated with an increased risk when assessed by different algorithms,^33^, ^34^, ^35^ these subjects more often have MACE at follow‐up, as shown in different Cox models adjusted for underlying morbidities and lifestyle. When other unconventional risk factors (i.e., reduced PDCU and partner sexual interest^36^, ^37^) were iteratively introduced in the Cox models, Δ age (M–F) still retained significance in predicting an unfavorable CV outcome. As previously stated, ED implies varying shades of microangiopathy and encompasses different unfavorable conditions that predispose to CV events. Besides standard CV risk factors, ED men are more prone to suffer from unconventional risks such as relational distress^20^ or reduced penile blood flow, which reflect the arterial health status^36^ and represent a potential alert for CV disease. On the other hand, a preserved partner's interest represents a protective factor for men, which favors more virtuous self‐care, a lower harmful lifestyle habit, and a more favorable healthy background.^9^, ^37^ We therefore categorized the sample according to whether or not the partner expressed sexual interest. In this scenario, the Δ age (M–F) negative effect is still present in those having a partner with high libido and is less evident in those without. The lack of protective effect by female sexual interest has probably multifactorial implications, including the stressful condition generated by a demanding pattern in front of ED men. Several relational items should be considered when facing an ageing male population that has relationships with younger mates.

Considering that the first quartile of Δ age includes couples with negative values (i.e., female older than the male partner), in a subanalysis we investigated the effect of having an older female mate on the overall relationship and on the male partner characteristics. After adjusting for confounders, we essentially found no major differences concerning the biochemical, hormonal, and sexual characteristics of the male partner. As expected, older female partners were more often in the menopausal state. In addition, a conflictual couple relationship is more often observed, that might deserve further investigation.

Several limitations should be recognized. First, all data related to the partner were derived from patients’ interviews and, therefore, from his perception, which, in some way, is otherwise psychologically relevant. The data were derived from a large cohort of patients reporting sexual dysfunctions and therefore cannot be generalized to those without or to the general population. The retrospective design of the study does not allow the definition of cause‐and‐effect relationships. However, all the analyses were adjusted for a great number of major confounders.

In conclusion, the detrimental effect of age on ED is not modified by younger partners with better sexual functioning. Although couples with an increased Δ age (M–F) represent a fruitful model for increasing couple fertility among those already fathers, they are overall demanding relationships where familial and other general stressors could negatively affect the whole health status, particularly on the CV side. As populations age, family dynamics are inherently intertwined with health issues. By emphasizing the nexus between andrological health status and age disparities within couples, this study enhances our comprehension of family life courses beyond sociodemographic research.

## AUTHOR CONTRIBUTIONS

Mario Maggi conceived the study. Mario Maggi and Clotilde Sparano performed the statistical analyses. Clotilde Sparano, Giulia Rastrelli, Giovanni Corona, Linda Vignozzi, Daniele Vignoli, and Mario Maggi drafted and approved the manuscript.

## CONFLICT OF INTEREST STATEMENT

The authors declare no conflict of interest.

## Data Availability

Data are available from the corresponding author, M.M., upon reasonable request.
